# Serum Krebs von den Lungen-6 level predicts disease progression in interstitial lung disease

**DOI:** 10.1371/journal.pone.0244114

**Published:** 2020-12-17

**Authors:** Ui Won Ko, Eun Jung Cho, Heung-Bum Oh, Hyun Jung Koo, Kyung-Hyun Do, Jin Woo Song

**Affiliations:** 1 Department of Pulmonary and Critical Care Medicine, Asan Medical Center, University of Ulsan College of Medicine, Seoul, Republic of Korea; 2 Department of Internal Medicine, Gachon University Gil Medical Center, Incheon, Republic of Korea; 3 Department of Laboratory Medicine, Asan Medical Center, University of Ulsan College of Medicine, Seoul, Republic of Korea; 4 Department of Radiology, Asan Medical Center, University of Ulsan College of Medicine, Seoul, Republic of Korea; Nippon Medical School, JAPAN

## Abstract

Disease progression (DP) in interstitial lung disease (ILD) is variable and difficult to predict. In previous reports, serum Krebs von den Lungen-6 (KL-6) was suggested to be useful in diagnosing and predicting survival in ILD. The aim of our study was to investigate the usefulness of serum KL-6 as a predictor of DP in ILD. Clinical data of 199 patients with ILD (idiopathic pulmonary fibrosis: 22.8%) were prospectively collected and serum KL-6 levels were measured. DP was defined as a relative decline in forced vital capacity (FVC) ≥ 10%, acute exacerbation, or death during follow-up. The median follow-up period was 11.1 months. The mean age of the subjects was 62.2 years, and 59.8% were male. DP occurred in 21.6% of patients. The progressed group showed lower FVC, lower diffusing capacity for carbon monoxide, lower the minimum oxygen saturation during the 6-minute walk test, higher fibrosis scores on high-resolution computed tomography, and higher KL-6 levels (826.3 vs. 629.0 U/mL; p < 0.001) than those of the non-progressed group. In receiver operating characteristic curve analysis, serum KL-6 levels were a significant predictor of DP in ILD (area under the curve = 0.629, p = 0.009, and the optimal cut-off level was 811 U/mL). In multivariable Cox analysis, high serum KL-6 levels (≥ 800 U/mL) were only independently associated with DP in ILD (HR 2.689, 95% CI 1.445–5.004, P = 0.002). Serum KL-6 levels might be useful to predict DP in patients with ILD.

## Introduction

Interstitial lung disease (ILD) is defined as a group of lung diseases affecting the interstitium and includes more than 200 different types, including idiopathic pulmonary fibrosis (IPF) [1]. The clinical course of ILD is highly variable and unpredictable; some patients appear stable or show a slow decline, whereas others show rapid deterioration or periods of relative stability interposed with periods of acute respiratory decline [2]. Thus, predicting disease progression is difficult but important for the effective management of ILD. Previous studies reported that older age, male sex, lower lung function (forced vital capacity [FVC], diffusing capacity of the lung for carbon monoxide [DL_CO_]), a usual interstitial pneumonia pattern, and more extensive disease on high-resolution computed tomography (HRCT) are associated with poor prognosis in ILD [3–9]. However, their predictive capacity may be limited by insufficient respiratory effort, complications such as emphysema or pulmonary hypertension, or interobserver variability [10–12].

Blood biomarkers are relatively easy to test and independent of patient effort or reader ability [13]. A number of blood biomarkers were reported to be useful in predicting diagnosis or prognosis in ILD, including surfactant proteins A (SP-A) and D (SP-D), monocyte chemoattractant proteins 1 (MCP-1) and 7 (MCP-7), chemokine ligand 18 (CCL-18), interleukin-8 (IL-8), and Krebs von den Lungen-6 (KL-6) [14–18]. KL-6 is a high-molecular-weight glycoprotein located on the surface of alveolar epithelial cells. Surface expression of KL-6 is induced during the regeneration of type II pneumocytes, and destruction of the air-blood barrier of affected lungs increases the permeability of KL-6, leading to increased blood concentration of KL-6 [19]. Thus, blood KL-6 is considered an indicator for pulmonary damage, and has been reported to be a useful biomarker for diagnosis and for estimating disease severity, acute exacerbation, and prognosis in ILD [20–22]. However, biomarkers for predicting disease progression of ILD are not well-defined. Therefore, the aim of this study was to investigate the role of KL-6 as a predictor for disease progression in ILD.

## Materials and methods

### Study population

**Fig 1:**
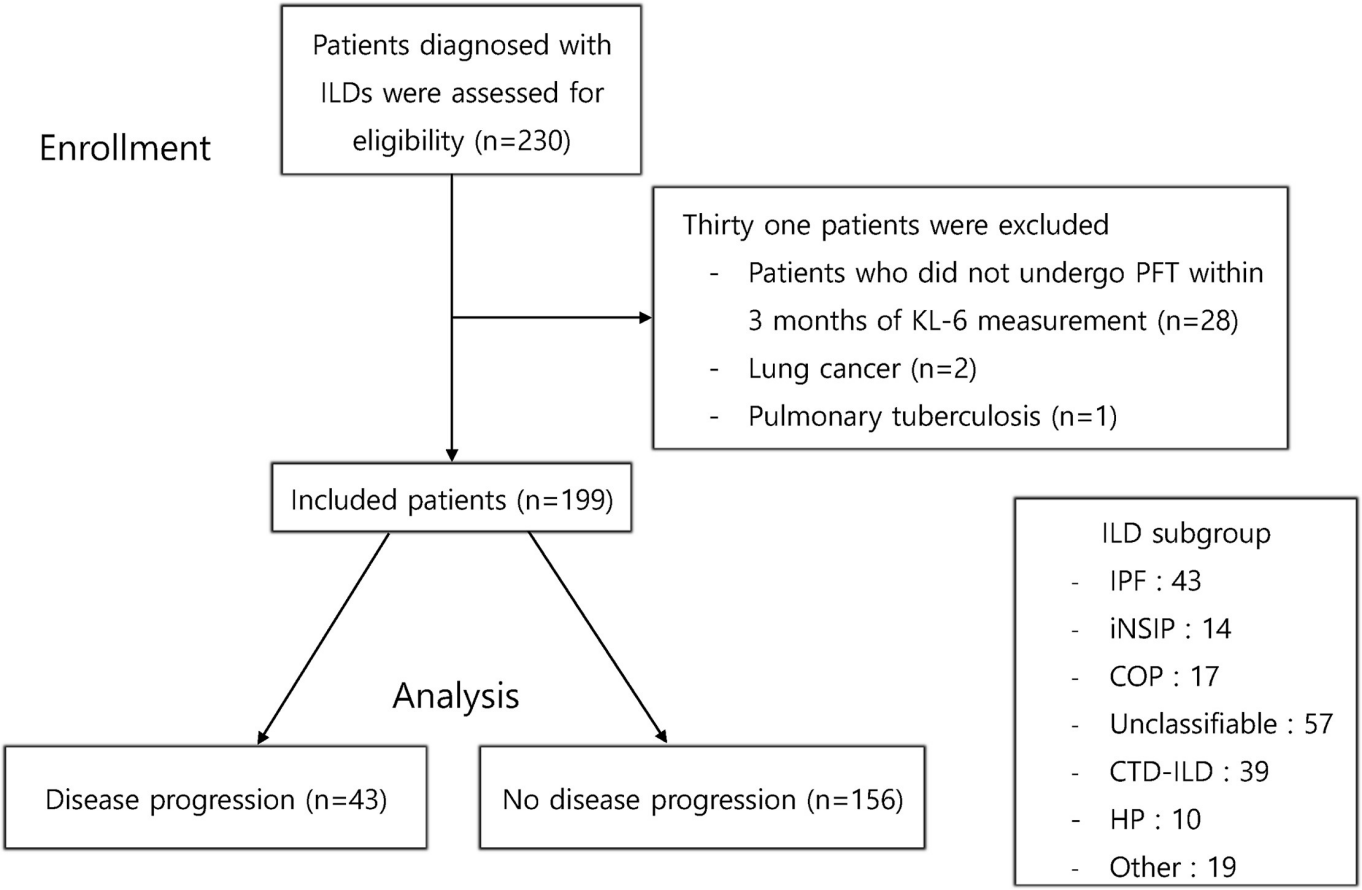
Flow chart of patient enrollment. KL-6, Krebs von den Lungen-6; N, number; ILD, interstitial lung disease; IPF, idiopathic pulmonary fibrosis; iNSIP, idiopathic non-specific interstitial pneumonia; COP, cryptogenic organizing pneumonia; Unclassifiable, unclassifiable interstitial lung diseases; CTD-ILD, connective tissue disease-associated interstitial lung disease; HP, hypersensitivity pneumonitis. The “other” group includes sarcoidosis, lymphangioleiomyomatosis, lymphocytic interstitial pneumonia, pneumoconiosis, eosinophilic pneumonia, IgG4-related disease, tuberous sclerosis complex-lymphangioleiomyomatosis, pulmonary alveolar proteinosis, respiratory bronchiolitis-interstitial lung disease, Langerhans cell histiocytosis, and pleuroparenchymal fibroelastosis.

From June to December 2016, 230 patients with ILD visited the ILD clinic at Asan Medical Center (Seoul, Republic of Korea) and were screened for this study. Among them, patients who did not undergo PFT within 3 months of KL-6 measurement and those with lung cancer or pulmonary tuberculosis were excluded in this study. Therefore, a total of 199 patients were included in this study (Fig 1). All patients underwent pulmonary function tests (PFT) and 197 underwent HRCT. Diagnosis of IPF, idopathic non-specific interstitial pneumonia (iNSIP), cryptogenic organizing pneumonia (COP) and unclassifiable ILD was made according to the international guidelines [23, 24]. Diagnosis of hypersensitivity pneumonitis (HP) was based on on histopathological findings (all biopsy-proven cases) [25]. Diagnosis of ILD in connective tissue disease (CTD-ILD) was confirmed based on the HRCT findings.

The study protocol was approved by the Institutional Review Board of Asan Medical Center (approval number 2016–0377) and written informed consent for the use of blood samples for clinical research was obtained from all patients.

### Clinical information

Clinical and survival data for all patients were prospectively collected, and all clinical parameters were obtained within 3 months of KL-6 measurement. Spirometry, total lung capacity (TLC) by plethysmography, and DL_CO_ were measured according to the recommendations, and results were expressed as percentages of normal predicted values [26–28]. Pulmonary function tests were performed every 3 months. The 6-minute walk test (6MWT) was performed according to the American Thoracic Society guidelines [29]. Acute exacerbation was defined according to the recommendation by Collard et al. [30] and disease progression was defined as a 10% or greater relative decline in FVC, acute exacerbation, or death during follow-up [23].

### HRCT evaluation

Two experienced chest radiologists (HJK and KHD), blinded to the clinical information, evaluated the HRCT images. HRCT findings were scored on a scale of 5% for all lobes, and classified based on a previous report by Ichikado et al., as follows: 1) normal attenuation, 2) ground-glass attenuation, 3) consolidation, 4) reticular abnormality, 5) traction bronchiectasis, and 6) honeycombing [31]. The fibrosis score (%) was calculated as the sum of honeycombing and reticulation scores, and the ILD extent (%) was the sum of ground-glass attenuation, reticulation, traction bronchiectasis, and honeycombing scores [32]. Disagreement between the two readers was resolved via a consensus. Representative HRCT images of IPF, CTD-ILD, COP, and unclassifiable ILD were presented in S1 Fig.

### Measurement of KL-6

Blood samples were obtained by venipuncture and were stored at -80°C until measurement. The serum levels of KL-6 were measured using an AU 5822 analyzer (Beckman Coulter, Brea, CA, USA) with the Nanopia KL-6 assay (Sekisui Medical, Tokyo, Japan). The KL-6 assay was performed using a latex-enhanced immunoturbidimetric assay method according to the manufacturer’s instructions.

### Statistical analysis

Student’s t-tests or Mann-Whitney U tests were used for continuous data and chi-square tests or Fisher’s exact tests were used for categorical data. Survival was estimated by Kaplan-Meier survival curves and compared by a log-rank test. Receiver operating characteristic (ROC) curve analysis was performed to confirm the optimal cut-off value of KL-6 for the prediction of disease progression. Risk factors were analyzed for disease progression with Cox proportional hazards models using backward elimination: variables with P < 0.1 in the univariate analysis were entered into the multivariable models. All *P* values were two-tailed, with statistical significance set at *P* < 0.05. All statistical analyses were performed using SPSS 21.0 (IBM, Armonk, NY).

## Results

### Baseline characteristics

The median follow-up period was 11.1 months (interquartile range [IQR], 10.0–13.0 months). For the 199 patients with ILD, the mean age was 62.2 years, and 59.8% were male. Moreover, 52.3% were ever-smokers (Table 1). The mean value of KL-6 was 671.6 U/mL (median: 487.5 U/mL, IQR: 319.5–801.5 U/mL). Among ILD cases, unclassifiable ILD was the most common (28.6%), followed by IPF (21.6%), connective tissue disease-associated interstitial lung disease (CTD-ILD; 19.6%), and cryptogenic organizing pneumonia (8.5%).

**Table 1:** Comparison of baseline characteristics between the IPF and non-IPF groups.

Characteristics	Total	IPF	Non-IPF	*P* value
No. of patients	199	43	156	
Age, years	62.2 (11.7)	66.0 (8.1)	61.1 (12.3)	0.002
Male	118 (59.8)	35 (81.3)	83 (53.2)	0.002
BMI, kg/m^2^	24.3 (3.2)	25.1 (2.5)	24.0 (3.3)	0.021
Ever-smoker	104 (52.3)	30 (69.8)	74 (47.4)	0.015
Smoking amount, pack-years	15.9 (21.1)	23.6 (21.2)	13.8 (20.6)	0.007
Interval between diagnosis and enrollment (years)	2.3 [1.2–5.0]	4.1 [2.0–6.8]	2.1 [1.1–4.6]	0.004
CRP, mg/L	0.5 (1.6)	0.8 (3.0)	0.4 (0.9)	0.400
KL-6, U/mL	671.6 (534.5)	877.2 (494.9)	614.9 (532.5)	0.004
FVC, % predicted	75.2 (18.2)	67.0 (15.5)	79.1 (17.4)	0.001
DL_CO_, % predicted	56.2 (18.4)	47.7 (13.9)	59.1 (18.4)	< 0.001
TLC, % predicted	76.3 (17.3)	66.1 (12.9)	77.4 (18.2)	< 0.001
6MWT distance, m	438.4 (92.4)	435.1(89.2)	439.4 (93.7)	0.796
6MWT, lowest SpO_2_, %	91.8 (4.6)	89.0 (5.0)	92.7 (4.1)	< 0.001
Bronchoalveolar lavage				
Neutrophils, %	9.9 (17.3)	9.7 (13.7)	9.9 (18.2)	0.936
Lymphocytes, %	21.4 (19.3)	13.2 (11.7)	23.6 (20.3)	< 0.001
HRCT, %				
Honeycombing	5.5 (8.8)	12.1 (11.0)	3.3 (6.6)	< 0.001
Reticular abnormality	10.3 (6.8)	12.1 (5.3)	9.7 (7.2)	0.019
Ground-glass attenuation	9.2 (10.6)	7.9 (7.1)	9.6 (11.6)	0.252
Traction bronchiectasis	6.9 (5.4)	9.4 (4.4)	6.0 (5.4)	< 0.001
Fibrosis score	15.8 (18.2)	24.2 (13.9)	13.0 (11.0)	< 0.001
ILD extent	34.0 (19.0)	43.8 (15.2)	30.7 (19.0)	< 0.001
Treatments				< 0.001
Steroid ± immunosuppressants	75 (37.7)	4 (9.3)	71 (45.5)	
Antifibrotics	39 (19.6)	32 (74.4)	7 (4.5)	
None	85 (42.7)	7 (16.3)	78 (50.0)	

Data are presented as mean (standard deviation), median [interquartile range] or number (%), unless otherwise indicated.

IPF, idiopathic pulmonary fibrosis; BMI, body mass index; CRP, C-reactive protein; KL-6, Krebs von den Lungen-6; FVC, forced vital capacity; DL_CO_, diffusing capacity of the lung for carbon monoxide; TLC, total lung capacity; 6MWT, 6-minute walk test; SpO_2,_ peripheral blood oxygen saturation; HRCT, high-resolution computed tomography; ILD, interstitial lung disease

The IPF group showed older age, more proportion of male subjects and ever-smokers, higher KL-6 levels, higher body mass index (BMI), lower lung function (FVC, DL_CO_, TLC), and the lowest oxygen saturation during the 6MWT than the non-IPF group (Table 1). The IPF group also had higher scores of honeycombing, reticulation, traction bronchiectasis, fibrosis, and ILD extent on HRCT compared with the non-IPF group.

### Correlations between KL-6 levels and disease severity

**Fig 2:**
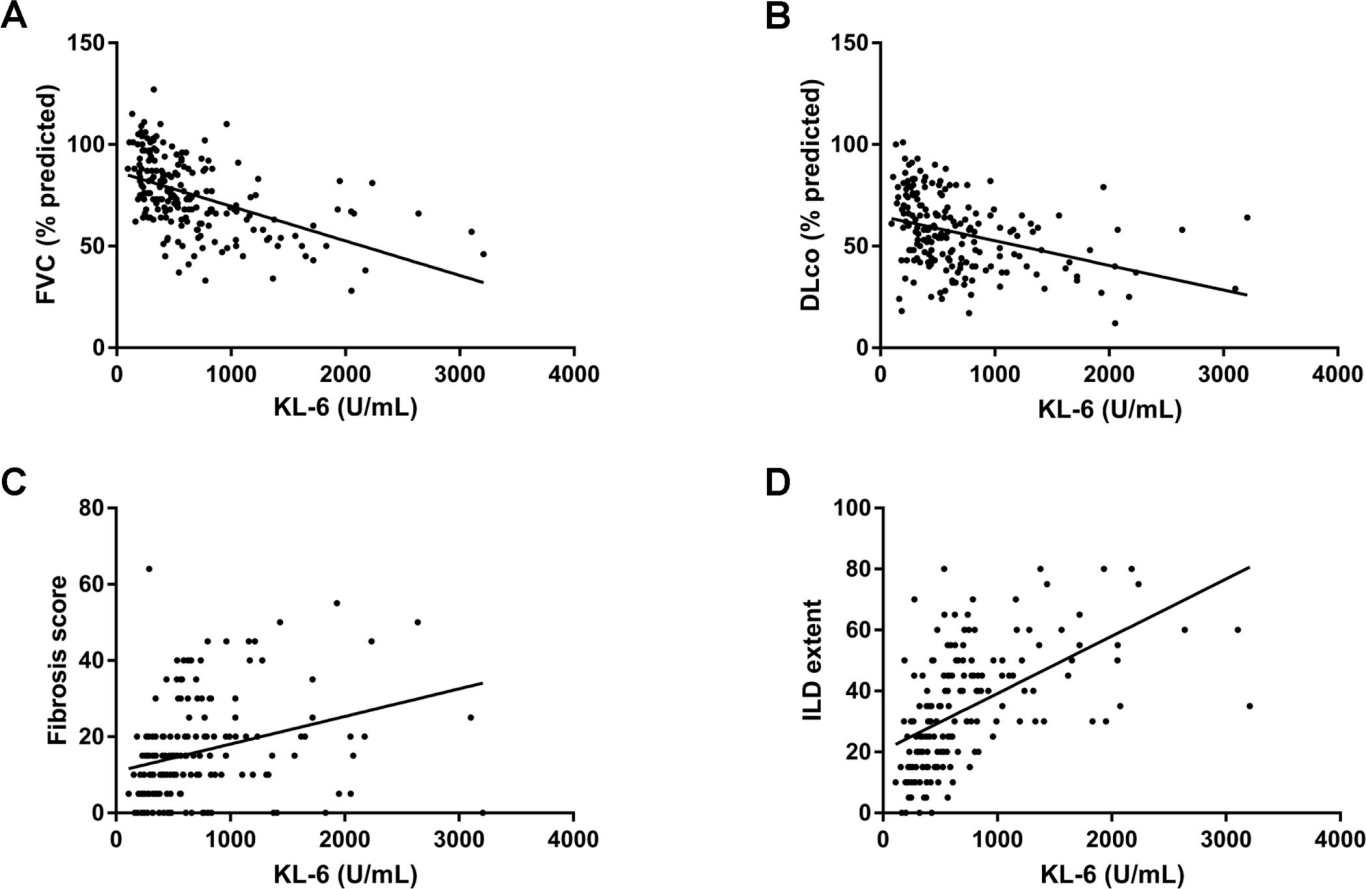
Correlation between KL-6 levels and disease severity. (A) Correlation between KL-6 level and FVC, (B) Correlation between KL-6 level and DLco, (C) Correlation between KL-6 level and fibrosis score, (D) Correlation between KL-6 level and ILD extent. KL-6, Krebs von den Lungen 6; FVC, forced vital capacity; DL_CO_, diffusing capacity of the lung for carbon monoxide; ILD, interstitial lung disease.

Serum KL-6 levels were inversely correlated with FVC (r = -0.27, *P* < 0.001) and DL_CO_ (r = -0.36, *P* < 0.001) (Fig 2A and 2B). Serum KL-6 levels were positively correlated with fibrosis scores (r = 0.33, *P* < 0.001) and ILD extent (r = 0.54, *P* < 0.001) on HRCT (Fig 2C and 2D).

### Predicting factors for disease progression

During follow-up, 43 patients (21.6%) experienced disease progression (acute exacerbation in 3 patients, decline in FVC ≥ 10% in 40 patients, and no death; the mean interval of PFT: 3 months). The progressed group had higher KL-6 levels, lower FVC, lower TLC, lower the minimum oxygen saturation during the 6MWT, and a higher ILD extent on HRCT than the non-progressed group (Table 2).

**Table 2:** Comparison of baseline characteristics between progressed and non-progressed groups.

Characteristics	DP	Non-DP	*P* value
No. of patients	43	156	
Age, years	64.2 (10.9)	61.6 (11.8)	0.206
Male	25 (58.1)	93 (59.6)	1.000
BMI, kg/m^2^	25.0 (3.5)	24.1 (3.1)	0.091
Ever-smoker	22 (51.2)	82 (52.6)	1.000
Smoking amount, pack-years	18.5 (24.2)	15.2 (20.2)	0.363
Interval between diagnosis and enrollment (years)	2.0 [1.1–5.2]	2.6 [1.2–4.8]	0.459
CRP, mg/L	0.8 (3.0)	0.4 (0.9)	0.369
KL-6, U/mL	826.3 (531.6)	629.0 (529.0)	0.032
FVC, % predicted	68.7 (17.5)	77.0 (18.0)	0.008
DL_CO_, % predicted	54.5 (17.0)	57.2 (18.4)	0.382
TLC, % predicted	69.1 (17.2)	78.2 (16.9)	0.002
6MWT distance, m	424.0 (95.4)	442.8 (91.4)	0.262
6MWT, lowest SpO_2_, %	89.9 (5.2)	92.4 (4.2)	0.002
Bronchoalveolar lavage			
Neutrophils, %	6.5 (9.8)	10.9 (18.9)	0.05
Lymphocytes, %	20.8 (17.1)	21.6 (20.0)	0.81
HRCT, %			
Honeycombing	6.4 (9.5)	5.2 (8.6)	0.459
Reticular abnormality	12.4 (8.3)	9.6 (6.2)	0.056
Ground-glass attenuation	11.8 (15.2)	8.4 (8.9)	0.193
Traction bronchiectasis	7.4 (4.8)	6.7 (5.5)	0.440
Fibrosis score	18.8 (13.8)	14.9 (12.3)	0.085
ILD extent	41.0 (18.0)	31.9 (18.8)	0.008
IPF	15 (34.9)	28 (17.9)	0.029
Treatments			< 0.001
Steroid ± immunosuppressants	17 (39.5)	58 (37.2)	
Antifibrotics	17 (39.5)	22 (14.1)	
None	9 (20.9)	76 (48.7)	

Data are presented as mean (standard deviation), median [interquartile range] or number (%).

DP, disease progression; BMI, body mass index; CRP, C-reactive protein; KL-6, Krebs von den Lungen-6; FVC, forced vital capacity; DL_CO_, percentage predicted diffusing capacity of the lung for carbon monoxide; TLC, total lung capacity; 6MWT, 6-minute walk test; BAL, bronchoalveolar lavage; SpO_2_, peripheral blood oxygen saturation; HRCT, high-resolution computed tomography; ILD, interstitial lung disease; IPF, idiopathic pulmonary fibrosis

**Fig 3:**
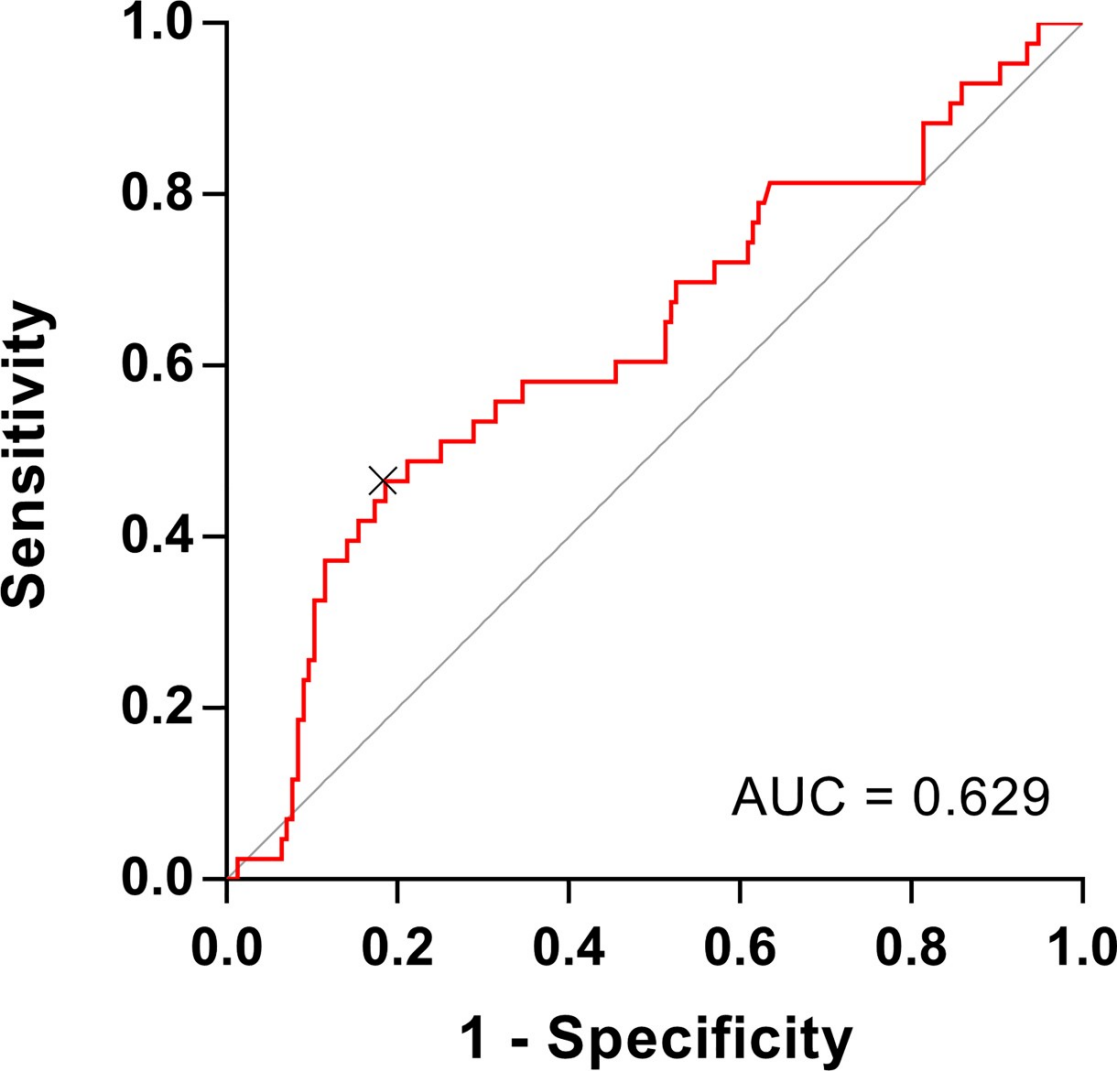
Receiver operating characteristic curve analysis of serum KL-6 level for disease progression in patients with ILD. KL-6, Krebs von den Lungen-6; ILD, interstitial lung disease; AUC, area under the curve.

In ROC curve analysis, serum KL-6 level was a significant predictor of disease progression in ILD, and the optimal cut-off value was 811 U/mL (area under the curve [AUC] = 0.629, *P* = 0.009, sensitivity of 46.5%, specificity of 81.4%) (Fig 3). In an unadjusted Cox proportional hazards model, high KL-6 levels (≥ 800 U/mL), lower lung function (FVC), and lower the minimum oxygen saturation during the 6MWT, diagnosis of IPF and use of antifibrotics, were significantly related to disease progression (Table 3). In the multivariable analysis including age, KL-6 level (≥ 800 U/mL), FVC, lowest oxygen saturation during 6MWT, and diagnosis of IPF, only KL-6 levels (≥ 800 U/mL) were the independent predictive factor for disease progression (HR 2.689, 95% CI 1.445–5.004, P = 0.002).

**Table 3:** Risk factors for disease progression in patients with ILD assessed by Cox regression analysis.

Variables	HR (95% CI)	*P* value
Unadjusted analysis		
Age, years	1.028 (0.999–1.058)	0.058
Male	0.939 (0.512–1.722)	0.839
BMI, kg/m^2^	1.076 (0.978–1.183)	0.132
Ever-smoker	1.082 (0.595–1.969)	0.796
CRP, mg/L	1.076 (0.963–1.203)	0.197
KL-6 (≥ 800 U/mL)	2.533 (1.389–4.618)	0.002
Interval between diagnosis and enrollment (years)	1.004 (0.905–1.114)	0.937
FVC, % predicted	0.980 (0.964–0.997)	0.019
DL_CO_, % predicted	0.995 (0.979–1.012)	0.562
Distance during 6MWT, m	0.998 (0.995–1.001)	0.238
Lowest oxygen saturation during 6MWT, %	0.928 (0.877–0.982)	0.010
BAL, Neutrophils, %	0.985 (0.956–1.015)	0.316
BAL, lymphocytes, %	1.000 (0.982–1.018)	0.965
Fibrosis score on HRCT, %	1.016 (0.993–1.039)	0.172
ILD subtype		
None-IPF (Ref.)	1	
IPF	2.115 (1.129–3.963)	0.019
Treatment*		0.002
None (Ref.)	1	
Antifibrotics	4.086 (1.813–9.209)	0.001
Steroid ± immunosuppressants	1.920 (0.849–4.344)	0.117
Multivariable analysis		
KL-6 (≥ 800 U/mL)	2.689 (1.445–5.004)	0.002

ILD, interstitial lung disease; BMI, body mass index; CRP, C-reactive protein; KL-6, Krebs von den Lungen-6; FVC, forced vital capacity; DL_CO_, percentage predicted diffusing capacity of the lung for carbon monoxide; 6MWT, 6-minute walk test; TLC, total lung capacity; BAL, bronchoalveolar lavage; HRCT, high-resolution computed tomography; IPF, idiopathic pulmonary fibrosis; Ref, reference

* Because treatment is highly related to ILD subtype (antifibrotic agents have been used mostly in IPF patients), treatment was not included in the multivariable analysis.

### Comparison of survival between low and high KL-6 groups

**Fig 4:**
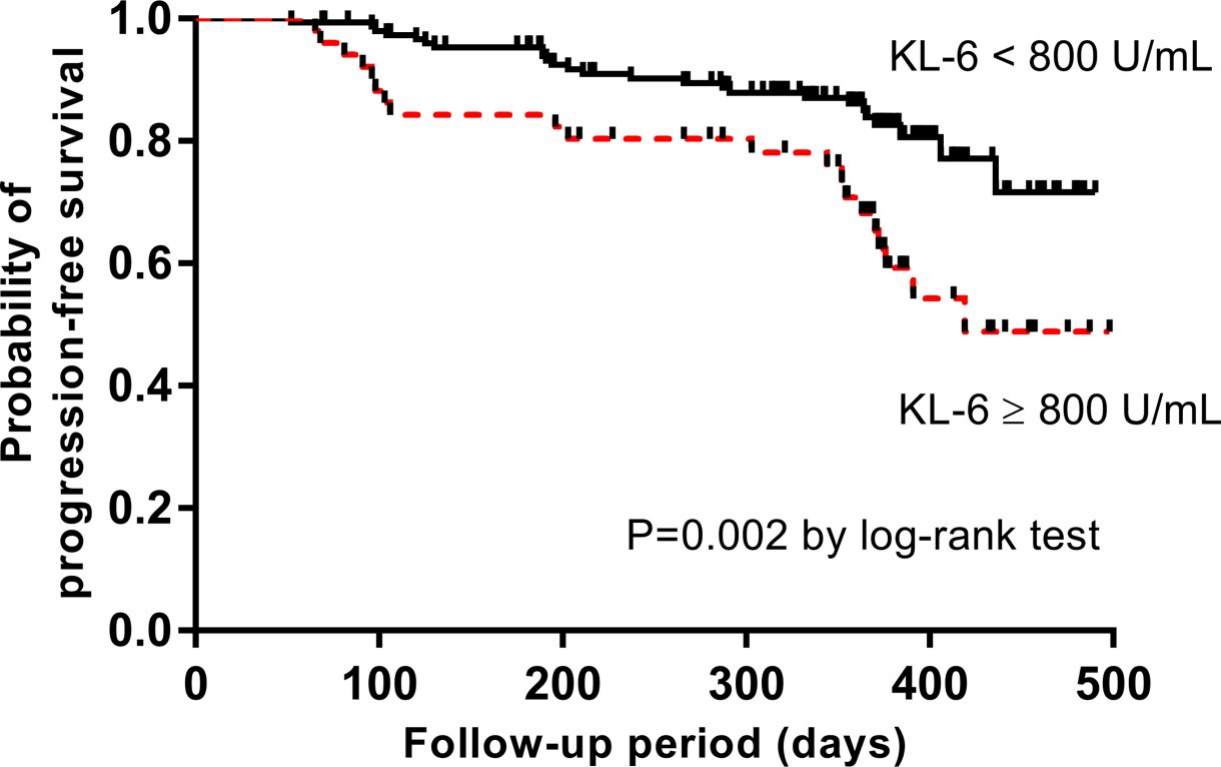
Comparison of survival curves between high and low KL-6 groups. KL-6, Krebs von den Lungen-6.

The high KL-6 group (≥ 800 U/mL) showed a higher BMI, lower lung function (FVC, DL_CO_, TLC), and poorer exercise capacity (the lowest oxygen saturation and distance during 6MWT) than the low KL-6 group (< 800 U/mL) (Table 4). The high KL-6 group also showed higher scores of honeycombing, reticulation, ground-glass opacity, traction bronchiectasis, fibrosis, and ILD extent compared to the low KL-6 group. The high KL-6 group had a significantly lower progression-free survival rate than the low KL-6 group (median survival period: 419 days vs. not reached, *P* = 0.002, Fig 4).

**Table 4:** Comparison of baseline characteristics between high and low KL-6 groups.

Characteristics	High KL-6^a^	Low KL-6^b^	*P* value
No. of patients	51	148	
Age, years	63.6 (10.0)	61.7 (12.2)	0.315
Male	31 (60.8)	87 (58.8)	0.932
BMI, kg/m^2^	25.6 (2.5)	23.8 (3.3)	< 0.001
Ever-smoker	29 (56.9)	75 (50.7)	0.548
Smoking amount, pack-years	17.5 (23.7)	15.3 (20.2)	0.522
CRP, mg/L	0.7 (2.8)	0.3 (1.0)	0.283
KL-6, U/mL	1388.7 (576.2)	424.6 (179.0)	< 0.001
Interval between diagnosis and enrollment (years)	2.1 [1.2–4.5]	2.4 [1.2–5.1]	0.451
FVC, % predicted	62.3 (15.5)	79.6 (16.9)	< 0.001
TLC, % predicted	61.8 (12.8)	81.1 (15.9)	< 0.001
DL_CO_, % predicted	47.4 (14.6)	59.8 (18.1)	< 0.001
6MWT distance, m	410.9 (88.8)	449.4 (91.8)	0.013
6MWT, lowest SpO_2_, %	88.6 (4.5)	93.1 (4.0)	< 0.001
Bronchoalveolar lavage			
Neutrophils, %	7.8 (10.6)	10.7 (19.6)	0.264
Lymphocytes, %	20.0 (18.9)	22.1 (19.6)	0.565
HRCT, %			
Honeycombing	9.0 (11.3)	4.1 (7.1)	0.007
Reticular abnormality	13.0 (8.3)	9.2 (5.8)	0.005
Ground-glass attenuation	13.6 (13.7)	7.5 (8.6)	0.005
Traction bronchiectasis	10.2 (5.1)	5.5 (4.9)	< 0.001
Fibrosis score	21.9 (15.3)	13.3 (10.7)	0.001
ILD extent	47.7 (15.6)	28.5 (17.5)	< 0.001
IPF	21 (41.2)	22 (14.9)	<0.001
Treatments			<0.001
None	8 (15.7)	77 (52.0)	
Antifibrotics	20 (39.2)	19 (12.8)	
Steroid ± immunosuppressants	23 (45.1)	52 (35.1)	

Data are presented as mean (standard deviation), median [interquartile range] or number (%), unless otherwise indicated.

KL-6, Krebs von den Lungen-6; BMI, body mass index; CRP, C-reactive protein; FVC, forced vital capacity; TLC, total lung capacity; DL_CO_, diffusing capacity of the lung for carbon monoxide; 6MWT, 6-minute walk test; SpO_2_, peripheral blood oxygen saturation; HRCT, high-resolution computed tomography; ILD, interstitial lung disease; IPF, idiopathic pulmonary fibrosis

a: KL-6 ≥ 800 U/mL

b: KL-6 < 800 U/mL

## Discussion

In our study, baseline serum KL-6 levels were significantly higher in patients who experienced disease progression during follow-up compared to those who remained stable. Moreover, a high baseline serum KL-6 level (≥800 U/mL) was an independent risk factor for disease progression in ILD. These findings indicate that KL-6 is a useful prognostic marker for disease progression in patients with ILD.

Our study showed that high serum KL-6 was an independent prognostic factor in ILD. In previous studies, serum KL-6 was also suggested as an indicator of disease activity or progression of ILD [22, 33]. A study of 77 patients with IPF by Ohshimo et al. demonstrated that patients who developed acute exacerbation (n = 13) had significantly higher baseline serum KL-6 levels (2528 ± 1645 U/mL vs. 1584 ± 1000 U/mL; *P* < 0.0001) than those without acute exacerbation, suggesting that serum KL-6 could be a predictive marker for disease progression in IPF [22]. A study of 14 patients with rapidly progressive IPF by Yokoyama et al. also demonstrated that KL-6 levels in patients who survived (n = 8) significantly decreased from 2661 ± 1178 U/mL to 2160 ± 910 U/mL (*P* < 0.05) at 1 week and to 1801 ± 899 U/mL (*P* < 0.05) at 3 weeks after weekly steroid pulse therapy [33].

Our study also showed that a serum KL-6 level of 811 U/mL was the most discriminatory cut-off value predicting disease progression in patients with ILD. Previous studies also reported similar findings [34, 35]. Among two studies performed in Japan, one study of 27 IPF patients identified a baseline serum KL-6 level of 1000 U/mL as the most discriminatory cut-off value in predicting mortality (sensitivity 90.0%, specificity 70.6%) [34]. The other study, with a larger group of 219 patients with idiopathic interstitial pneumonias (IIPs) and CTD-ILD, also identified a baseline serum KL-6 level of 1000 U/mL as the most discriminatory cut-off value in predicting mortality (sensitivity 67.2%, specificity 60.2%) [35]. Moreover, another study of 77 IPF patients showed that a serum KL-6 level of 1300 U/mL was useful to predict the acute exacerbation of IPF (AUC = 0.736, sensitivity 92%, specificity 61%, *P* = 0.008) [22].

In our study, serum KL-6 levels showed a positive correlation with lung function or ILD extent on HRCT. Previous studies also reported that serum KL-6 levels were positively correlated with the disease severity of ILDs [36–38]. In a study of 101 patients with sarcoidosis by Honda et al., patients with elevated KL-6 levels (mean 802.4 U/mL) had significantly more frequent parenchymal involvement (ground-glass opacities, nodules, interlobular septal thickening, traction bronchiectasis, architectural distortion, and bronchial wall thickening) on chest CT than those with normal KL-6 levels (mean 305.7 U/mL) [37]. A study of 47 patients with rheumatoid arthritis-associated pulmonary disease by Kinoshita et al. also reported a positive correlation between serum KL-6 levels and total CT scores (r = 0.83, *P* < 0.001) [38]. In addition, a study of 98 patients with ILD by Qin et al. showed that serum KL-6 levels were significantly correlated with DL_CO_ (r = -0.513, *P* < 0.001) and CT scores (r = 0.539, *P* = 0.000) [39]. These results support that serum KL-6 levels may correlate with the severity of ILDs.

There are some limitations in this study. First, this was a study conducted in a single tertiary referral center. Further prospective multicenter studies are needed to confirm our findings. Second, the median follow-up period was relatively short. However, disease progression occurred in one-fifth of the patients, which was sufficient to confirm the significance of predicting disease progression by KL-6 levels. Finally, we analyzed heterogenous ILD patients together due to small numbers in each subgroup. However, in our previous study, KL-6 levels were not different between ILD subtypes [40].

## Conclusion

Baseline serum KL-6 levels are useful in predicting disease progression in patients with ILD. Larger-scale prospective studies are needed to confirm these findings.

## Supporting information

S1 Fig Representative HRCT images of ILD patients. (A) IPF (B) CTD-ILD (C) COP (D) unclassifiable ILD. HRCT, high-resolution computed tomography; ILD interstitial lung disease; IPF, idiopathic pulmonary fibrosis; CTD-ILD, connective tissue disease-associated interstitial lung disease, COP, cryptogenic organizing pneumonia.
(PPTX)
