# Supplementary material for: Serum Krebs von den Lungen-6 level predicts disease progression in interstitial lung disease
Source: PLoS One. 2020 Dec 17;15(12):e0244114. doi: 10.1371/journal.pone.0244114 (PMC7746162; doi:10.1371/journal.pone.0244114)
Supplement: S1 Fig — (A) IPF (B) CTD-ILD (C) COP (D) unclassifiable ILD. HRCT, high-resolution computed tomography; ILD interstitial lung disease; IPF, idiopathic pulmonary fibrosis; CTD-ILD, connective tissue disease-associated interstitial lung disease, COP, cryptogenic organizing pneumonia. (PPTX) [file pone.0244114.s001.pptx]

## Slide 1
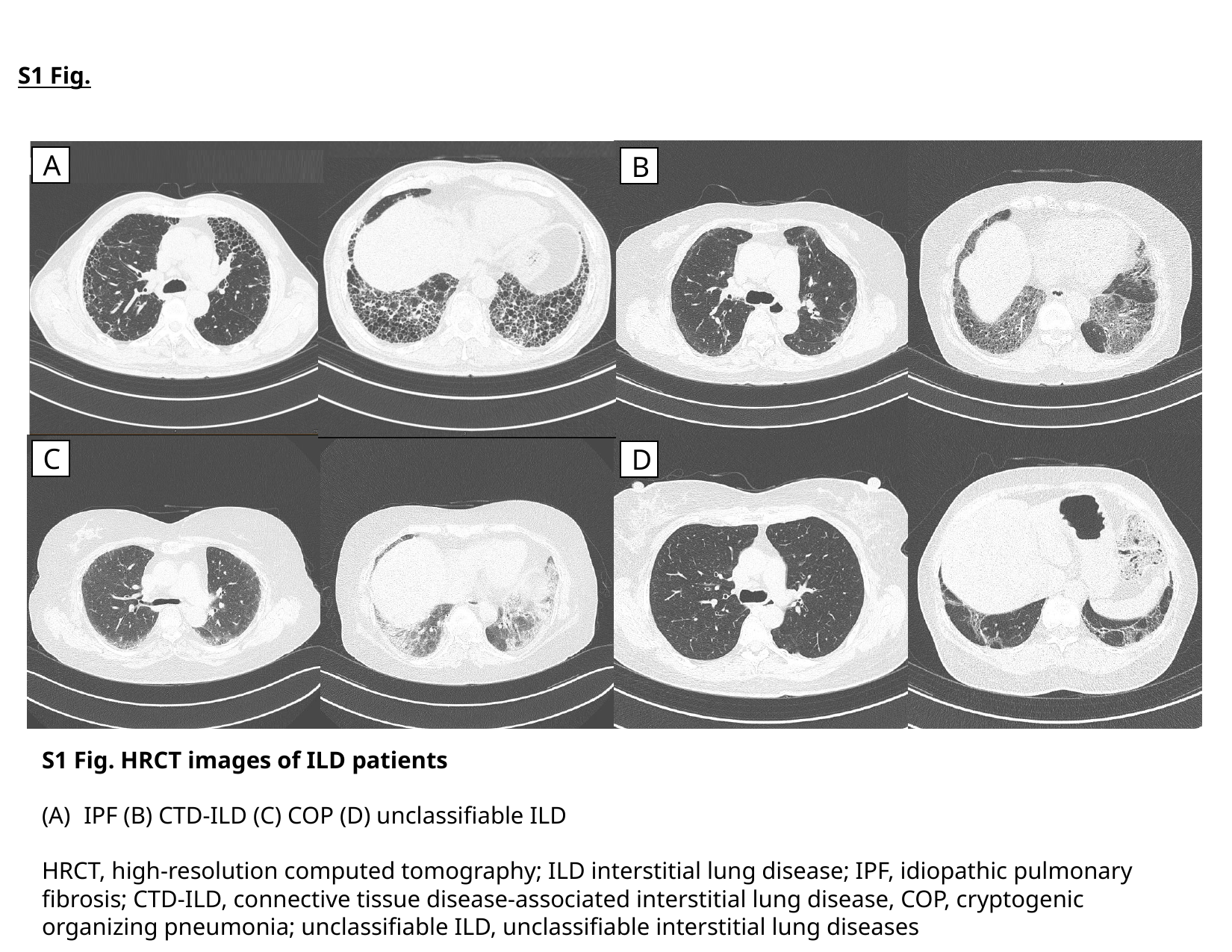

S1 Fig.
A
B
C
D
S1 Fig. HRCT images of ILD patients
IPF (B) CTD-ILD (C) COP (D) unclassifiable ILD
HRCT, high-resolution computed tomography; ILD interstitial lung disease; IPF, idiopathic pulmonary fibrosis; CTD-ILD, connective tissue disease-associated interstitial lung disease, COP, cryptogenic organizing pneumonia; unclassifiable ILD, unclassifiable interstitial lung diseases
